# Estimating the global and regional burden of meningitis in children caused by *Haemophilus influenzae* type b: A systematic review and meta-analysis

**DOI:** 10.7189/jogh.12.04014

**Published:** 2022-03-05

**Authors:** Jay J Park, Sandhya Narayanan, Jakov Tiefenbach, Ivana Lukšić, Boni Maxime Ale, Davies Adeloye, Igor Rudan

**Affiliations:** 1 Edinburgh Medical School, University of Edinburgh, 49 Little France Crescent, Edinburgh, UK; 2 School of Biological Sciences, University of Edinburgh, Grant Institute Kings Buildings, W Mains Rd, Edinburgh, UK; 3 Department of Microbiology, Teaching Institute of Public Health “Dr Andrija Štampar”, Zagreb, Croatia; 4 Holo Healthcare Limited, Nairobi, Kenya; 5 Centre for Global Health, Edinburgh Medical School, Usher Institute, University of Edinburgh, Edinburgh, Scotland, UK

## Abstract

**Background:**

*Haemophilus influenzae* Type B (Hib) meningitis caused significant public health concern for children. Recent assessment in 2015 suggests vaccination has virtually eliminated invasive Hib diseases. However, many countries launched their programs after 2010, and few are yet to establish routine Hib immunisations. We therefore aimed to update the most recent global burden of Hib meningitis before the impact of COVID-19 pandemic, from 2010 to 2020, in order to aid future public health policies on disease management and prevention.

**Methods:**

Epidemiological data regarding Hib meningitis in children <5 years old were systematically searched and evaluated from PubMed and Scopus in August, 2020. We included studies published between 2010 and 2019 that reported incidence, prevalence, mortality, or case-fatality-ratio (CFR), and confirmation of meningitis by cerebrospinal fluid culture, with a minimum one year study period and ten cases. Each data was stratified by one study-year. Median study-year was used if information was not available. Quality of all studies were assessed using our adapted assessment criteria from Grading of Recommendations Assessment, Development and Evaluation (GRADE) and Study Quality Assessment Tool for Observational Cohort and Cross-Sectional Studies from National Heart, Lung and Blood Institute (NHLBI). We constructed and visually inspected a funnel plot of standard error by the incidence rate and performed an Egger’s regression test to statistically assess publication bias. To ascertain incidence and CFR, we performed generalised linear mixed models on crude individual study estimates. Heterogeneity was assessed using I-squared statistics whilst further exploring heterogeneity by performing subgroup analysis.

**Results:**

33 studies were identified. Pooled incidence of global Hib meningitis in children was 1.13 per 100 000-child-years (95% confidence interval (CI) = 0.80-1.59). Southeast Asian Region (SEAR) of World Health Organisation (WHO) region reported the highest incidence, and European Region (EUR) the lowest. Considering regions with three or more data, Western Pacific Region (WPR) had the highest incidence rate of 5.22 (95% CI = 3.12-8.72). Post-vaccination incidence (0.67 cases per 100 000-child-years, 95% CI = 0.48-0.94) was dramatically lower than Pre-vaccination incidence (4.84 cases per 100 000-child-years, 95% CI = 2.95-7.96). Pooled CFR in our meta-analysis was 11.21% (95% CI = 7.01-17.45). Eastern Mediterranean Region (EMR) had the highest CFR (26.92, 95% CI = 13.41-46.71) while EUR had the lowest (4.13, 95% CI = 1.73-9.54). However, considering regions with three or more data, African Region (AFR) had the highest CFR at 21.79% (95% CI = 13.65-32.92). Before the coronavirus disease 2019 (COVID-19) impact, the estimation for global Hib meningitis cases in 2020 is 7645 and 857 deaths.

**Conclusions:**

Global burden of Hib meningitis has markedly decreased, and most regions have implemented vaccination programs. Extrapolating population-at-risk from studies has possibly led to an underestimation. Continuous surveillance is necessary to monitor vaccination impact, resurgence, vaccine failures, strain variance, COVID-19 impact, and to track improvement of regional and global Hib meningitis mortality.

*Haemophilus influenzae *(Hi) is a small, gram-negative, coccobacillary bacterium which frequently infects humans. Most Hi strains contain a polysaccharide capsule, and can be further classified by their capsular antigens into 6 important serotypes (ie, a through f). Hib has historically been the cause of more than 95% of Hi associated infections, most frequently affecting infants and children under the age of five [1]. An invasive Hib infection is diagnosed when the pathogen is detected in primary sterile tissues and bodily fluids (ie, blood, cerebrospinal fluid). It most commonly presents as pneumonia or meningitis, but other patterns of infection (ie, septicaemia, septic arthritis, osteomyelitis, pericarditis, cellulitis and epiglottitis) are also recognised [2].

Pneumonia and meningitis caused by Hib in young children presented a significant public health concern before the implementation of Hib immunisation programmes. In 2000, it was estimated that there were approximately 8.13 million cases of severe infections, and over 371 000 deaths in the population under the age of five [3]. Furthermore, in the unvaccinated population, Hib was the leading cause of non-epidemic bacterial meningitis during first year of life. Even with prompt and appropriate antibiotic treatment, mortality from Hib meningitis is still high (3%-20%). This can be significantly higher in countries where access to medical care is limited, and severe neurological sequelae can be seen in as many as 30%-40% of survivors [4].

With vaccines directly targeting the capsular antigen type b, invasive disease caused by Hib has virtually disappeared. The most recent assessment of Hib meningitis in 2015 by Walh et al., suggests that the number of children affected worldwide has fallen to 31 400 (13,400-50,800). However, it also comments on countries with low vaccine-coverage (ie, India, Nigeria, China, South Sudan, Russia, Thailand) accounting for the majority of deaths. They also reported a global CFR (Case-Fatality-Ratio) from Hib meningitis to be 19% (7%-29%), with Africa having the highest CFR of 61% (20%-98%). Despite the burden being disproportionately high in regions with lack of vaccination programs or with newly introduced vaccinations, continuous efforts in evaluating the burden of Hib meningitis are critical for policymakers to maintain or aim for complete elimination of the disease in their respective regions [5].

This systematic review aims to assess the available evidence from 2010 to 2019 on the incidence of Hib meningitis in children under the age of five within the six main World Health Organisation (WHO) regions, to generate updated estimates of the global burden for 2020, up to the outbreak of COVID-19 pandemic. Consistent monitoring of Hib meningitis incidence is crucial in reducing disease burden and prompting legislators to develop appropriate disease-prevention strategies. We also aim to provide insights into methodological difficulties and improvements for future studies in this field.

## METHODS

We adapted our previous approach to estimate the global burden of meningitis in children [6]. The review was conducted in line with the PRISMA guidelines (Table S1 in the **Online Supplementary Document**) [7].

### Data sources

The search strategy was defined to capture all possibly informative studies while excluding the majority of the irrelevant studies. No studies were excluded based on the language of publication. The full text of the studies was retrieved wherever possible.

The search strategy used was:

“meningitis AND (incidence OR prevalence OR mortality OR morbidity OR sequela* OR case fatality OR risk factor*)”

and was applied as a free search in the title/abstract/keywords field in both PubMed and Scopus databases for publications between 01 January 2010 and 01 January 2020 (Table S2 in the **Online Supplementary Document**). The search was conducted on 15 August 2020.

### Eligibility criteria

Meningitis was defined as an acute bacterial disease with sudden onset of fever, intense headache, nausea, vomiting, neck stiffness [6]. Clinical presentations from the International Classification of Diseases, Tenth Revision (ICD-10) and WHO (Table S3 in the **Online Supplementary Document**) were applied in our quality assessment criteria [8].

Studies were included if:

1. Reported incidence, prevalence, mortality or case-fatality-ratio (CFR) for Hib meningitis (studies had to specify or have clear suggestions that *Hi* was referring to serotype B)
2. Confirmed meningitis by cerebrospinal fluid (CSF) culture in facility-based laboratory.
3. Conducted a minimum study period of one year (or multiples of one year).
4. Reported minimum 10 cases of confirmed Hib meningitis during study period.
5. Defined age group to 0-4.
6. Contained no methodological ambiguities about how the study was conducted, systematic error in the design of research that could affect the results of the study.

### Data extraction

Titles were screened by two independent authors (JJP, JT) and finalised studies were those that met all the inclusion criteria. Disagreement with inclusion or exclusion was settled by a third author (DA). We emailed authors in order to retrieve full texts that were not available online or through the University of Edinburgh library.

We extracted the following from all retrievable studies for data analysis: Study period, WHO region, study site, study design, study duration, study setting, income level of study setting, vaccination era, diagnostic criteria, age range, number of cases and deaths confirmed during study period, population denominator, incidence, and CFR (Table S4 in the **Online Supplementary Document**). Data was stratified by a study period of one-year, if possible. Median study year was extracted from the study periods where information for each study-year was not available. Any of these unavailable or ambiguous in text were clarified by contacting authors of the studies via email.

### Quality assessment and risk of bias

No standardised assessment tools exist for observational studies. Therefore, we adapted the Grading of Recommendations Assessment, Development and Evaluation (GRADE) and Study Quality Assessment Tool for Observational Cohort and Cross-Sectional Studies from National Heart, Lung and Blood Institute (NHLBI) (Table S5 in the **Online Supplementary Document**). Quality was assessed by two independent authors (JJP, SN) and disagreement was settled by a third author (DA).

### Statistical analysis

For studies that did not have a defined population at risk, data was extrapolated from published censuses [9]. For population in each region not defined for age 0-4, data was extrapolated by multiplying the ratio of age 0-4 population of the whole country. Data for calculating the ratio was uniformly extracted from the World Population Prospects 2019, Department of Economic and Social Affairs, United Nations.

Incidence was standardised to reflect the number of new cases per-100,000-person-years; however, this was not age-standardised due to lack of age-specific stratification of data.

### Meta-analysis

We performed a generalised linear mixed models (GLMMs) meta-analysis on crude individual study estimate. Assuming a binomial (or Poisson) distribution, standard errors were determined from the reported crude estimates and population denominators.

We constructed and visually inspected a funnel plot of standard error by the incidence rate and performed an Egger’s regression test to statistically assess publication bias. Heterogeneity between studies was assessed using I-squared (*I^2^*) statistics. We further explored heterogeneity by performing subgroup analysis on the following:

- Study design (prospective, retrospective) and Study setting (hospital, community-based).
- WHO region: To estimate and compare the global burden of Hib meningitis in different regions.
- Income level: To determine whether the income level of the countries affects the burden of Hib meningitis.
- Study period: To compare the studies that were conducted before and after 2010.
- Vaccination period: Data grouped into post- and pre-vaccination periods to quantify effect of vaccination program on Hib meningitis.
- Study quality: Sensitivity analysis based on the risk of bias.

All analyses were done using R (version 4.1.0, R foundation for Statistical Computing, Vienna, Austria) with packages dmetar, meta, and metafor.

## RESULTS

### Search result

**Figure 1:**
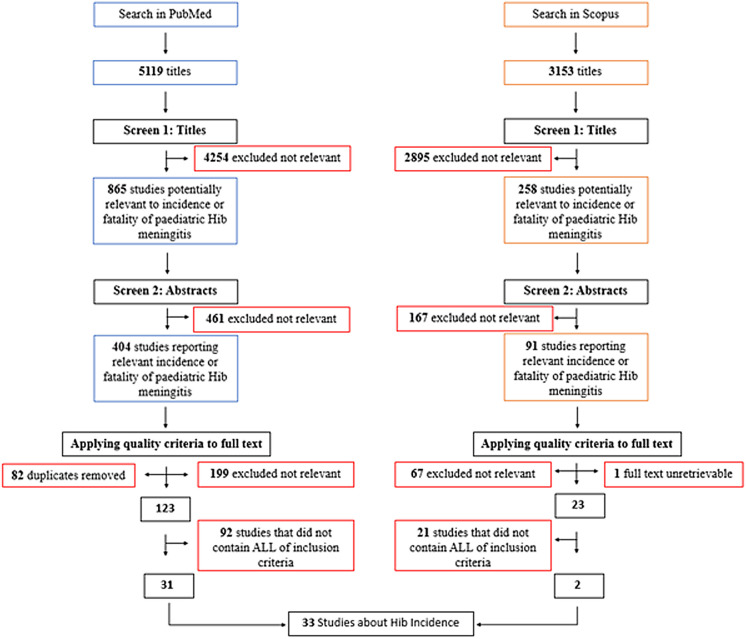
Flowchart of literature search about *Haemophilus influenzae* type b (Hib) incidence.

Databases returned 8272 searches in total (PubMed 5119 and Scopus 3153). 404 studies from PubMed and 91 studies from Scopus were remaining after screening for title and abstract. 82 duplicates were removed. Inclusion criteria were applied to exclude 199 studies from PubMed and 67 studies from Scopus searches. 1 full text from Scopus was unretrievable. We further excluded 113 studies that did not contain all our inclusion criteria and selected 33 studies for the review (**Figure 1**).

### Study characteristics

33 studies were spread across each of the six WHO regions (Table S6 in the **Online Supplementary Document**). Both AFR and WPR had the most study outputs of nine articles. There were five articles from EUR, four from EMR, three from American (AMR), and two articles from SEAR. 21 papers were rated as high, 11 papers of moderate, and 1 paper of low quality. The funnel plot showed some degree of data symmetry (standard error by incidence rate of Hib meningitis), shown by weak evidence against Egger’s test (-1.93, *P* = 0.057), suggesting no publication bias (Figure S1 in the **Online Supplementary Document**).

### Incidence

**Figure 2:**
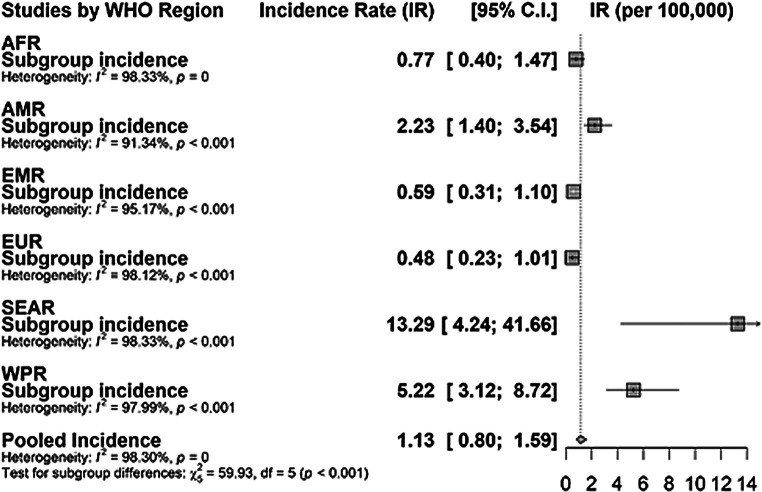
Pooled incidence of *Haemophilus influenzae* type b (Hib)meningitis by WHO regions

**Figure 3:**
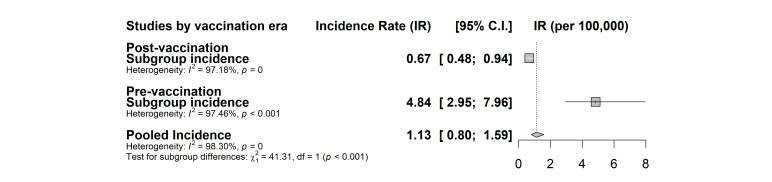
Pooled incidence of *Haemophilus influenzae* type b (Hib)meningitis by vaccination period.

Following meta-analysis, the pooled incidence of global Hib meningitis for underage of five was 1.13 per 100 000 child-years (95% confidence interval (CI) = 0.80-1.59) with high *I^2^* heterogeneity of 98.3% (*P* < 0.001). In our WHO regional subgroup analysis, EUR had the smallest incidence rate of 0.48 (0.23-1.00) cases with *I^2^* of 98.1% (*P* < 0.001). SEAR had the highest incidence rate of 13.29 (4.24-41.66) with *I^2^* of 98.3% (*P* < 0.001). However, considering studies that have three or more data, WPR had the highest incidence rate of 5.22 (95% CI = 3.12-8.72) (**Figure 2**). We also assessed the impact of the vaccination program by comparing the pre-vaccination incidence (4.84 cases per 100 000 child-years, 95% CI = 2.95-7.96) and the post-vaccination incidence (0.67 cases per 100 000 child-years, 95% CI = 0.48-0.94) (**Figure 3**). Low-middle-income countries (LMICs) had the lowest incident rate of 0.52 cases per 100 000 child-years (95% CI = 0.31-0.86) compared to that of High-income countries (HICs) that had 2.24 cases per 100,00 child-years (95% CI = 0.94-5.30) (Figure S2 in the **Online Supplementary Document**). Hospital-based studies had a higher incidence rate of 2.04 cases per child-years (95% CI = 1.35-3.09) compared to community-based studies which had 0.5 cases per child-years (95% CI = 0.33-0.82, *P* < 0.001) (Figure S2 in the **Online Supplementary Document**). Furthermore, studies that were conducted before 2010 (2.02 cases per 100 000 child-years, 95% CI = 1.43-2.86) had a significantly higher incident rate compared to those conducted after 2010 (0.37 cases per 100 000 child-years, 95% CI = 0.20-0.68) (Figure S2 in the **Online Supplementary Document**).

**Figure 4:**
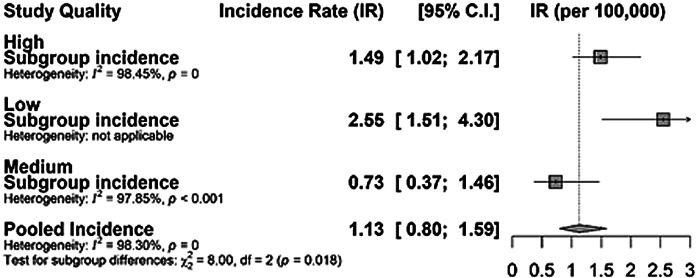
Sensitivity analysis between quality of studies and incidence rate of *Haemophilus influenzae* type b (Hib)meningitis.

Finally, when studies were compared in terms of their quality, estimates from high-quality studies and the overall global estimates were comparable, which further attests to the internal validity of our estimates (**Figure 4**).

### CFR

**Figure 5:**
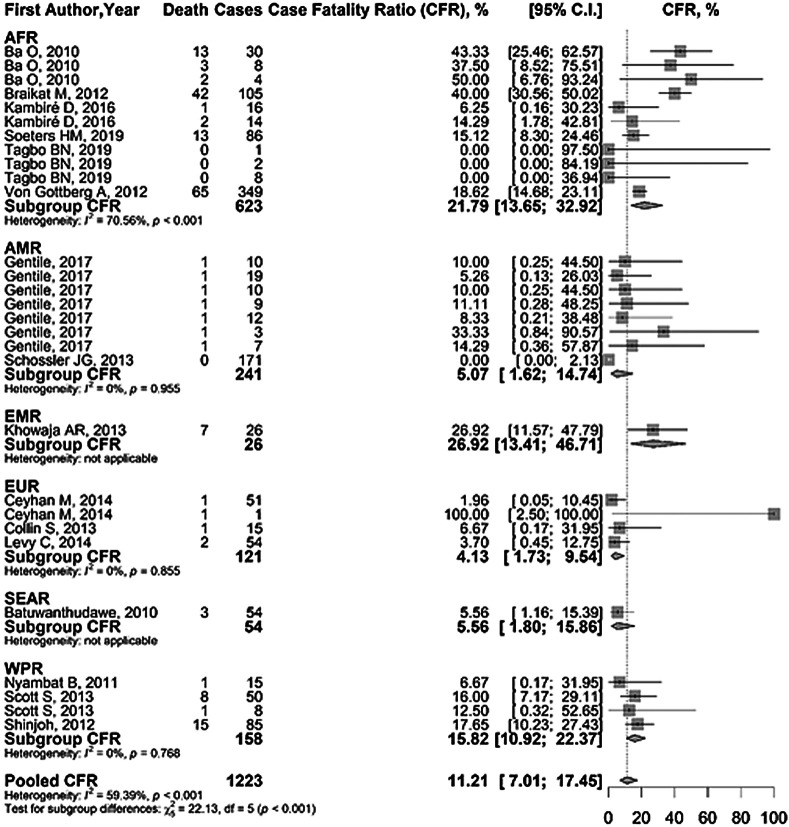
Case fatality ratio (CFR) of *Haemophilus influenzae* type b (Hib)meningitis by WHO regions.

Pooled CFR in our meta-analysis was 11.21% (95% CI = 7.01-17.45) with heterogeneity (*I^2^*) of 59.4% (*P* < 0.001). Our subgroup analysis of WHO regions (*P* < 0.001) showed that EMR had the highest CFR (26.92%, 95% CI = 13.41-46.71) and EUR had the lowest CFR (4.13%, 95% CI = 1.73-9.54). However, considering studies with three or more data, AFR had the highest CFR at 21.79% (**Figure 5**). LICs had the largest CFR of 18.82% (95% CI = 11.72-28.81, *P* = 0.022). LMICs had the lowest CFR of 3.37% (95% CI = 0.27-30.98, *P* = 0.016) closely followed by HICs with 8.93% (95% CI = 3.34-21.75, *P* = 0.062). There was no increase in CFR with the increase in the incidence rate.

Our incidence rate was applied to the world population of children under the age of five from the World Population Prospects 2019 and we estimated the global cases of Hib meningitis of children under the age of five in 2020 to be 7645 and 857 deaths.

## DISCUSSION

We provide a crude-assessment of the most recent global burden of Hib meningitis in children under the age of five without vaccine, aetiology-specific, or Human Immunodeficiency Virus (HIV)-prevalence adjustments [6]. This global, regional, and national Hib burden estimation is based on observed cases, to provide estimates compatible with former approximations. Such differences in methodology account for the marginal discrepancies between our findings and the previous evaluations of Hib meningitis [3,5].

Challenges in accurately estimating the burden still persist [6]. Longitudinal epidemiological studies are crucial in establishing disease burden and traditionally, these have been particularly lacking in developing countries where surveillance data are most needed. Specific regions reported having limited population-based information on paediatric disease burden were AFR, SEAR, EMR, and China – regions which contain the largest population of children [10]. We encountered a similar difficulty, acquiring the least number of data sets from SEAR and EMR. It is however noteworthy that AFR had the greatest number of data sets included in this report. Such contribution is attributable to the increase in surveillance networks such as the Paediatric Bacterial Meningitis Surveillance Network in WHO African Region, WHO’s Invasive Bacterial Vaccine-preventable Diseases Surveillance Network, MenAfriNet, and other nationwide and hospital-based surveillances [11-13]. One of the factors that contributed to the lack of data was studies either failing to specify the type of *Hi* or lacking resources to differentiate serotypes. Due to the nature of the disease, many patients were reported to have taken antibiotics prior to collection of CSF, reducing chances of bacterial isolation and leading to underestimation [14].

**Figure 6:**
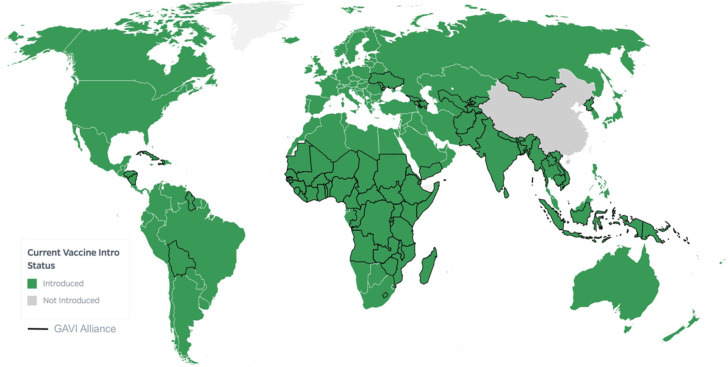
World *Haemophilus influenzae* type b (Hib)vaccination status [15].

The reduction in the Hib meningitis burden is evident in literature [2,3,5,13]. Our estimates for the global burden have markedly decreased compared to that of 2015 [5]. A study in 2005 reported AFR to have the highest incidence rate [3]. However, our results reflect a dramatic decrease in the incidence rate in AFR, which were in line with the estimation of 2015 [5]. Multi-regression analysis showed a strong correlation between vaccination and incidence rates; therefore, such successful elimination of Hib meningitis can be attributed to the vaccination status of AFR. EUR and AFR are the only regions that fully implemented vaccination programs in all countries (**Figure 6**). Amongst the multiple factors that resulted in the success, the Global Alliance for Vaccines and Immunizations (GAVI) has been pivotal in overcoming the barrier to access [16]. Although the highest incidence was reported from WPR followed by SEAR in the previous estimation, our result showed SEAR to have a disproportionately high incidence rate. Due to previously observed disease resurgence, the standard time-range to determine the vaccine effectiveness is at least 5 years post-introduction [17]. Interestingly, 70% of the vaccinated countries in SEAR implemented the vaccination program after 2010 which might have contributed to the phenomenon (Table S7 in the **Online Supplementary Document**). Moreover, SEAR had the least number of studies included in this study, limiting the exact estimation. Accurate assessment of the vaccination impact and the burden of Hib meningitis in SEAR warrants continuous monitoring efforts. In WPR, it is a problem that China is yet to include Hib vaccine in the national immunisation program [15]. Hib vaccine in China is voluntary and a recent meta-analysis shows that the coverage is very low [18]. Disparity has been shown especially in the central and west regions of China which are less developed than the eastern provinces [18].

Walh et al. adjusted the CFR due to CFR accounting for mortality of those having access to medical care or study sites [7]. Therefore, the unadjusted CFR in this study may be an underestimation. In line with Walh’s report, AFR had the highest CFR followed by EMR, while EUR had the lowest. However, SEAR and AMR had comparably low CFRs whilst CFR in WPR was high, in contrast to the estimation in 2015. This variability may be explained by the lack of data: AMR, EMR, SEAR, and WPR all had less than three studies included for CFR calculation. As stated numerously in literature, a consistently high CFR in AFR can be associated with the inadequate state of health care infrastructure throughout the region: AFR still has the leading mortality rate for children under five [13,19,20].

### Limitations

There were few limitations in this study. Although we included papers across all the six WHO regions, the paucity of data in many settings should guide interpretation. We reported unadjusted incidence rates which in some instances may not be representative, drawing limitations on true burden of the disease. This limitation has been described in previous studies, with authors describing their estimates as minimum due to the limitation of identifying cases in the population [21,22]. Our sub-group analysis of incidence stratified by income level of countries should therefore also be interpreted with caution. Studies performed in low-resource settings report the difficulty in ascertaining the true incidence [23,24].

Furthermore, we adapted the GRADE and NHLBI approach to quality in the absence of a standardised tool for evaluating the quality of observational epidemiological studies [25,26]. Extrapolating the denominator through censuses, using ratios to estimate the population of a certain region, could have also affected our estimates, as this could have potentially increased the denominator, leading to an underestimation of the incidence rate. (Table S8 in the **Online Supplementary Document**). Moreover, there is an intrinsic limitation to many passive sentinel surveillance systems which are subject to hospitals that have referrals from areas outside the surveillance regions, incomplete case identification, and the discrepancy in medical access in rural areas [27,28]. Some studies combined surveillance data of several countries without noting the catchment population of each centre.

Additionally, not all studies were stratifiable into individual study years and we had to use the median-study-year in order to categorise the data set into pre- vs post-vaccination period and before vs after 2010. Not all population-based studies reported the mortality rate or the number of deaths, leading to a limited data set to calculate global and regional CFR. This is reflected in our subgroup analysis on income level of countries vs CFR, where HICs and LMICs had less than ten data included for analysis.

### Implications for policy and practice

Despite the near elimination of Hib meningitis in most regions, evidence strongly supports the need for continuous surveillance systems. Investing in the long-term monitoring of meningitis is crucial in detecting its epidemiology and evaluating the effectiveness of vaccination programs [10]. Such efforts give valuable regional data to focus policies in establishing self-funded vaccine programs, once GAVI funding ends [27]. Furthermore, collecting data gives policymakers evidence-based motivation to direct budgets and implement policies to maintain low infection rates. In Sri Lanka, the lack of Hib infection data compromised the importance of Hib disease despite its impeccable public health record [29]. Ultimately, our data set also reflects that more abundance of surveillance data will be required to increase the accuracy of the estimation of global, regional, and national burden of Hib meningitis in children [30].

There is no doubt that population-based surveillance of Hib meningitis confirmed by laboratory findings is the most reliable method in providing data for estimation of Hib burden [31]. However, such a time-consuming approach can be difficult in countries with limited resources. Therefore, WHO developed a standardised rapid assessment tool (RAT). It was used to estimate Hib meningitis in AFR and EMR which reported similar approximations to population-based studies in the respective regions [32]. Further studies have testified to the consistency and credibility of Hib RAT, recommending its use for regions where conditions are unfavourable for laboratory-based surveillance [33].

Future estimations should account for HIV infections and its impact on Hib diseases. HIV infection can cause immunosuppression that exposes one to a greater risk of attaining an encapsulated bacterial infection such as Hib [34,35]. Although evidence shows there is only a slight increase in risk of attaining Hib meningitis for HIV-infected children compared to those who are not infected, severity is markedly increased [20]. Not to mention, monitoring is important in keeping track of vaccine failures that are more prevalent in populations with high rates of HIV [13,36,37]. Lastly, a thorough appraisal of vaccine program successes in regions like AFR is recommended, in order to apply the same principles in countries without a vaccine program and for establishing policies for other endemic diseases.

With significant reduction of Hib infections following the implementation of the vaccine, the paradigm has shifted to non-type b Hi becoming the most common Hi infection [38]. Although no evidence confirms the replacement of the type b strain due to vaccination, dominance of a and f serotypes have been observed in particular regions [39-41]. Hi type a is especially notable since it can cause similar invasive disease to Hib [42]. In addition, Nontypeable *Haemophilus influenzae* (NTHi) has replaced Hib as the most common isolate causing invasive *Hi* disease. Its importance has been shunned previously because it was easily treated with beta-lactam antibiotics; however, evidence supports an increase in resistance to antibiotics [43,44]. Furthermore, NTHi has a high level of genetic diversity, hindering effective vaccine development. Continuous monitoring of *Hi* disease is therefore important in investigating strain variations, clinical impact, and vaccine development [45].

This study marks an important milestone in assessing the global burden of Hib meningitis in children, as our results reflect the ultimate assessment before COVID-19 pandemic. The pandemic has impacted the epidemiology of many infectious diseases including Hib. A recent report warned against an increase in Hib disease burden in under-five-year-olds due to a decline in the childhood vaccination rate during the pandemic, despite COVID-19 protective measures [46]. As this study was based on a prediction model with its own limitations and the pandemic is still ongoing, future efforts to monitor and conduct epidemiological studies on Hib meningitis are crucial. Furthermore, the WHO has also reported that an increasing number of children missed their vaccines in 2020, especially in SEAR and EMR [47]. Undoubtedly, this study establishes its role as a comparative measure when assessing COVID-19 impact on global burden of Hib meningitis in children under the age of five.

## CONCLUSIONS

Hib-conjugate-vaccine programs have nearly alleviated the global burden of Hib meningitis in children. Yet, continuous surveillance is pivotal in measuring the impact of vaccinations, monitoring resurgence and vaccine failures, strain variance, and tracking the improvement of Hib meningitis mortality both regionally and globally. We also strongly recommend future surveillance projects to report the respective catchment population as well as establishing centres that are generalisable to the entire population. Most importantly, this assessment provides the most up-to-date data on Hib meningitis in children before the COVID-19 pandemic, which can be compared to evaluations during and post-pandemic era, to determine the effect of the pandemic on the epidemiology of Hib disease.

## Additional material


Online Supplementary Document
