# Supplementary material for: Estimating the global and regional burden of meningitis in children caused by Haemophilus influenzae type b: A systematic review and meta-analysis
Source: J Glob Health. 2022 Mar 5;12:04014. doi: 10.7189/jogh.12.04014 (PMC8893283; doi:10.7189/jogh.12.04014)
Supplement: Online Supplementary Document [file jogh-12-04014-s001.pdf]

Table S1: PRISMA 2020 Checklist

Section and Topic	Item #	Checklist item	Location where item is reported
**TITLE**			
Title	1	Identify the report as a systematic review.	Pg 0
**ABSTRACT**			
Abstract	2	See the PRISMA 2020 for Abstracts checklist.	Pg 1
**INTRODUCTION**			
Rationale	3	Describe the rationale for the review in the context of existing knowledge.	Pg 1
Objectives	4	Provide an explicit statement of the objective(s) or question(s) the review addresses.	Pg 1
**METHODS**			
Eligibility criteria	5	Specify the inclusion and exclusion criteria for the review and how studies were grouped for the syntheses.	Pg 2
Information sources	6	Specify all databases, registers, websites, organisations, reference lists and other sources searched or consulted to identify studies. Specify the date when each source was last searched or consulted.	Pg 2
Search strategy	7	Present the full search strategies for all databases, registers and websites, including any filters and limits used.	Pg 2
Selection process	8	Specify the methods used to decide whether a study met the inclusion criteria of the review, including how many reviewers screened each record and each report retrieved, whether they worked independently, and if applicable, details of automation tools used in the process.	Pg 2-3
Data collection process	9	Specify the methods used to collect data from reports, including how many reviewers collected data from each report, whether they worked independently, any processes for obtaining or confirming data from study investigators, and if applicable, details of automation tools used in the process.	Pg 2-3
Data items	10a	List and define all outcomes for which data were sought. Specify whether all results that were compatible with each outcome domain in each study were sought (e.g. for all measures, time points, analyses), and if not, the methods used to decide which results to collect.	Pg 2-3
	10b	List and define all other variables for which data were sought (e.g. participant and intervention characteristics, funding sources). Describe any assumptions made about any missing or unclear information.	Pg 3
Study risk of bias assessment	11	Specify the methods used to assess risk of bias in the included studies, including details of the tool(s) used, how many reviewers assessed each study and whether they worked independently, and if applicable, details of automation tools used in the process.	Pg 3
Effect measures	12	Specify for each outcome the effect measure(s) (e.g. risk ratio, mean difference) used in the synthesis or presentation of results.	Pg 3-4
Synthesis methods	13a	Describe the processes used to decide which studies were eligible for each synthesis (e.g. tabulating the study intervention characteristics and comparing against the planned groups for each synthesis (item #5)).	Pg 3
	13b	Describe any methods required to prepare the data for presentation or synthesis, such as handling of missing summary statistics, or data conversions.	Pg 3
	13c	Describe any methods used to tabulate or visually display results of individual studies and syntheses.	Pg 3
	13d	Describe any methods used to synthesize results and provide a rationale for the choice(s). If meta-analysis was performed, describe the model(s), method(s) to identify the presence and extent of statistical heterogeneity, and software package(s) used.	Pg 3-4
	13e	Describe any methods used to explore possible causes of heterogeneity among study results (e.g. subgroup analysis, meta-regression).	Pg 3-4
	13f	Describe any sensitivity analyses conducted to assess robustness of the synthesized results.	Pg 3
Reporting bias assessment	14	Describe any methods used to assess risk of bias due to missing results in a synthesis (arising from reporting biases).	-
Certainty assessment	15	Describe any methods used to assess certainty (or confidence) in the body of evidence for an outcome.	Pg 3
**RESULTS**			
Study selection	16a	Describe the results of the search and selection process, from the number of records identified in the search to the number of studies included in the review, ideally using a flow diagram.	Pg 4
	16b	Cite studies that might appear to meet the inclusion criteria, but which were excluded, and explain why they were excluded.	-
Study characteristics	17	Cite each included study and present its characteristics.	Supplementary 3
Risk of bias in studies	18	Present assessments of risk of bias for each included study.	Pg 4
Results of individual studies	19	For all outcomes, present, for each study: (a) summary statistics for each group (where appropriate) and (b) an effect estimate and its precision (e.g. confidence/credible interval), ideally using structured tables or plots.	Pg 4-5
Results of syntheses	20a	For each synthesis, briefly summarise the characteristics and risk of bias among contributing studies.	Pg 4
	20b	Present results of all statistical syntheses conducted. If meta-analysis was done, present for each the summary estimate and its precision (e.g. confidence/credible interval) and measures of statistical heterogeneity. If comparing groups, describe the direction of the effect.	Pg 4-5
	20c	Present results of all investigations of possible causes of heterogeneity among study results.	Pg 4-5
	20d	Present results of all sensitivity analyses conducted to assess the robustness of the synthesized results.	Pg 4-5
Reporting biases	21	Present assessments of risk of bias due to missing results (arising from reporting biases) for each synthesis assessed.	-
Certainty of evidence	22	Present assessments of certainty (or confidence) in the body of evidence for each outcome assessed.	Pg 4-5
**DISCUSSION**			
Discussion	23a	Provide a general interpretation of the results in the context of other evidence.	Pg 5-7
	23b	Discuss any limitations of the evidence included in the review.	Pg 7
	23c	Discuss any limitations of the review processes used.	Pg 7
	23d	Discuss implications of the results for practice, policy, and future research.	Pg 7-8
**OTHER INFORMATION**			
Registration and protocol	24a	Provide registration information for the review, including register name and registration number, or state that the review was not registered.	Pg 2
	24b	Indicate where the review protocol can be accessed, or state that a protocol was not prepared.	-
	24c	Describe and explain any amendments to information provided at registration or in the protocol.	-
Support	25	Describe sources of financial or non-financial support for the review, and the role of the funders or sponsors in the review.	Pg 0
Competing interests	26	Declare any competing interests of review authors.	Pg 0
Availability of data, code and other materials	27	Report which of the following are publicly available and where they can be found: template data collection forms; data extracted from included studies; data used for all analyses; analytic code; any other materials used in the review.	-

Table S2: Search Query for Literature

Database	Search Query

PubMed	(("meningeal"[All Fields] OR "meninges"[MeSH Terms] OR "meninges"[All Fields] OR "meninge"[All Fields] OR "meningism"[MeSH Terms] OR "meningism"[All Fields] OR "meningisms"[All Fields] OR "meningitis"[MeSH Terms] OR "meningitis"[All Fields] OR "meningitides"[All Fields]) AND ("epidemiology"[MeSH Subheading] OR "epidemiology"[All Fields] OR "incidence"[All Fields] OR "incidence"[MeSH Terms] OR "incidences"[All Fields] OR "incident"[All Fields] OR "incidents"[All Fields] OR ("epidemiology"[MeSH Subheading] OR "epidemiology"[All Fields] OR "prevalence"[All Fields] OR "prevalence"[MeSH Terms] OR "prevalance"[All Fields] OR "prevalences"[All Fields] OR "prevalence s"[All Fields] OR "prevalent"[All Fields] OR "prevalently"[All Fields] OR "prevalents"[All Fields]) OR ("mortality"[MeSH Terms] OR "mortality"[All Fields] OR "mortalities"[All Fields] OR "mortality"[MeSH Subheading]) OR ("epidemiology"[MeSH Subheading] OR "epidemiology"[All Fields] OR "morbidity"[All Fields] OR "morbidity"[MeSH Terms] OR "morbid"[All Fields] OR "morbidities"[All Fields] OR "morbids"[All Fields]) OR "sequela*"[All Fields] OR (("ieee int conf automation sci eng case"[Journal] OR "case phila"[Journal] OR "case"[All Fields]) AND ("fatal"[All Fields] OR "fatalities"[All Fields] OR "fatality"[All Fields] OR "fatally"[All Fields])) OR (("risk"[MeSH Terms] OR "risk"[All Fields]) AND "factor*"[All Fields]))) AND ((humans[Filter]) AND (allchild[Filter] OR allinfant[Filter] OR infant[Filter] OR newborn[Filter] OR preschoolchild[Filter]) AND (2010:2020[pdat]))
Scopus	TITLE-ABS-KEY (meningitis AND (incidence OR prevalence OR mortality OR morbidity OR sequela* OR case AND fatality OR risk AND factor*)) AND (LIMIT-TO (PUBYEAR , 2019) OR LIMIT-TO (PUBYEAR , 2018) OR LIMIT-TO (PUBYEAR , 2017) OR LIMIT-TO (PUBYEAR , 2016) OR LIMIT-TO (PUBYEAR , 2015) OR LIMIT-TO (PUBYEAR , 2014) OR LIMIT-TO (PUBYEAR , 2013) OR LIMIT-TO (PUBYEAR , 2012) OR LIMIT-TO (PUBYEAR , 2011) OR LIMIT-TO (PUBYEAR , 2010)) AND (LIMIT-TO (EXACTKEYWORD , "human") OR LIMIT-TO (EXACTKEYWORD , "humans"))

Table S3: WHO definition of Acute meningitis*

**Suspected Meningits for Case Finding**
• Any child aged 0-59 months admitted to hospital with sudden onset fever (>38.5˚C rectal or 38˚C axillary) and one of the following signs: neck stiffness, altered consciousness with no other alternative diagnosis, or other meningeal signs OR<br>• Any patient aged 0-59 months hospitalised with a clinical diagnosis of meningitis
**Probable Bacterial Meningitis**
A suspected meningitis case with cerebrospinal fluid (CSF) examination showing at least one of the following:<br>• Turbid appearance<br>• Leucocytosis (>100 cells/mm3)<br>• Leucocytosis (10-100 cells/mm3) AND either an elevated protein (> 100 mg/dL) or deceased glucose (< 40 mg/dL). Note: If protein and glucose results are not available, diagnosis using the first two conditions (turbid appearance or leucocytosis > 100 cells/mm3)
**Confirmed H. Influenzae Meningitis**
A suspected or probable meningitis case that is laboratory-confirmed by culture or identification of H. Influenzae (by antigen detection, immunochromotography, polymerase chain reaction (PCR) or other methods) in the CSF or blood from a child with a clinical syndrome consistent with meningitis.

Haemophilus influenzae [Internet]. Who.int. 2018 [cited 17 July 2021]. Available from: https://www.who.int/immunization/monitoring_surveillance/burden/vpd/WHO_SurveillanceVaccinePreventable_05_HaemophilusInfluenzae_R2.pdf?ua=1

Table S4: Data Extraction

Author/Year of Publication	Study Period	Median Study Year	Study Era	WHO Region	Study Site	Study Design	Study Duration (Mo)	Study Setting	Vaccination Era	Vaccine Introduction Year	Diagnostic Criteria	Age Range (mo)	Number of Cases (Confirmed All;PRO YEAR)	Number of Deaths (Confirmed All;PRO YEAR)	Population Denominator	Incidence (per 100,000; Age ≤5)	Case-Fatality Rate (CFR)	Quality
Ba O et al., 2010	2006	2006	0	AFR	Senegal	1	12	2	2	2005	BC, LA, CSF, B	0-59	30.00	12.86	1,312,300.00	2.2861	0.42857143	9
Ba O et al., 2010	2007	2007	0	AFR	Senegal	1	12	2	2	2005	BC, LA, CSF, B	0-59	8.00	3.43	1,312,300.00	0.6096	0.42857143	9
Ba O et al., 2010	2008	2008	0	AFR	Senegal	1	12	2	2	2005	BC, LA, CSF, B	0-59	4.00	1.71	1,312,300.00	0.3048	0.42857143	9
Ba O et al., 2010	2002	2002	0	AFR	Dakar	1	12	2	1	2005	BC, LA, CSF, B	0-59	41.00	NP	269,900.00	15.1908	NP	9
Ba O et al., 2010	2003	2003	0	AFR	Dakar	1	12	2	1	2005	BC, LA, CSF, B	0-59	30.00	NP	269,900.00	11.1152	NP	9
Ba O et al., 2010	2004	2004	0	AFR	Dakar	1	12	2	1	2005	BC, LA, CSF, B	0-59	56.00	NP	269,900.00	20.7484	NP	9
Ba O et al., 2010	2005	2005	0	AFR	Dakar	1	6	2	1	2005	BC, LA, CSF, B	0-59	24.00	NP	269,900.00	8.8922	NP	9
Ba O et al., 2010	2005	2005	0	AFR	Dakar	1	6	2	2	2005	BC, LA, CSF, B	0-59	16.00	NP	269,900.00	5.9281	NP	9
Ba O et al., 2010	2006	2006	0	AFR	Dakar	1	12	2	2	2005	BC, LA, CSF, B	0-59	12.00	NP	269,900.00	4.4461	NP	9
Ba O et al., 2010	2007	2007	0	AFR	Dakar	1	12	2	2	2005	BC, LA, CSF, B	0-59	2.00	NP	269,900.00	0.7410	NP	9
Ba O et al., 2010	2008	2008	0	AFR	Dakar	1	12	2	2	2005	BC, LA, CSF, B	0-59	1.00	NP	269,900.00	0.3705	NP	9
Edmond et al., 2010	2009	2009	0	AFR	Dakar, Senegal	1	12	1	2	2005	CSF, LA	1-59	24.00	NP	312,300.00	7.68491835	NP	7
Kaboré NF et al., 2012	2004-2005	2005	0	AFR	Burkina Faso (Bobo Dioulasso)	2	24	2	1	2006	CSF, PCR, BC, LA, G	0-59	47.00	NP	166,109.81	28.2945357	NP	7
Kaboré NF et al., 2012	2006-2008	2007	0	AFR	Burkina Faso (Bobo Dioulasso)	2	36	2	2	2006	CSF, PCR, BC, LA, G	0-59	27.00	NP	280,515.46	9.62513792	NP	7
Kambiré D et al., 2016	2011	2011	1	AFR	Burkina Faso	1	12	1	2	2006	CSF, PCR, BC, LA, G	0-59	16.00	1.22	1,600,000.00	1	0.07625	10
Kambiré D et al., 2016	2012	2012	1	AFR	Burkina Faso	1	12	1	2	2006	CSF, PCR, BC, LA, G	0-59	13.00	0.00	1,625,000.00	0.8	0	10
Kambiré D et al., 2016	2013	2013	1	AFR	Burkina Faso	1	12	1	2	2006	CSF, PCR, BC, LA, G	0-59	14.00	1.64	2,333,333.33	0.6	0.11714286	10
Karou SD et al., 2012	2007-2010	2009	0	AFR	Togo	2	48	2	1 + 2	2008	CSF, BC, G	0-59	14.00	NP	549,534.77	2.54760948	NP	3
Mwenda JM et al., 2019	2011	2011	1	AFR	South East Africa (Ethiopia, Lesotho, Madagascar, Kingdom of Eswatini, Swaziland	1	12	1	2	2007	PCR. CSF, S	1-59	1.00	NP	4644803.678	0.02152944	NP	6

					, Uganda, Zambia, Zimbabwe													
Mwenda JM et al., 2019	2012	2012	1	AFR	South East Africa (Ethiopia, Lesotho, Madagascar, Kingdom of Eswatini, Swaziland, Uganda, Zambia, Zimbabwe	1	12	1	2	2007	PCR. CSF, S	1-59	9.00	NP	4703752.899	0.19133658	NP	6
Mwenda JM et al., 2019	2013	2013	1	AFR	South East Africa (Ethiopia, Lesotho, Madagascar, Kingdom of Eswatini, Swaziland, Uganda, Zambia, Zimbabwe	1	12	1	2	2007	PCR. CSF, S	1-59	10.00	NP	4943658	0.20227936	NP	6
Mwenda JM et al., 2019	2014	2014	1	AFR	South East Africa (Ethiopia, Lesotho, Madagascar, Kingdom of Eswatini, Swaziland, Uganda, Zambia, Zimbabwe	1	12	1	2	2007	PCR. CSF, S	1-59	11.00	NP	5106123.716	0.2154276	NP	6
Mwenda JM et al., 2019	2015	2015	1	AFR	South East Africa (Ethiopia, Lesotho, Madagascar, Kingdom of Eswatini, Swaziland, Uganda, Zambia, Zimbabwe	1	12	1	2	2007	PCR. CSF, S	1-59	10.00	NP	5246857.624	0.19059027	NP	6
Mwenda JM et al., 2019	2016	2016	1	AFR	South East Africa (Ethiopia, Lesotho, Madagascar, Kingdom of Eswatini, Swaziland, Uganda, Zambia, Zimbabwe	1	12	1	2	2007	LA, PCR. CSF, S	1-59	12.00	NP	5400276.569	0.22221084	NP	6
Mwenda JM et al., 2019	2011	2011	1	AFR	West-Central Africa (Benin, Cameroon, Gambia, Ghana, Niger, Nigeria, Senegal, Togo)	1	12	1	2	2007	LA, PCR. CSF, S	1-59	0.00	NP	2123693.9	0	NP	6
Mwenda JM et al., 2019	2012	2012	1	AFR	West-Central Africa (Benin, Cameroon, Gambia, Ghana, Niger, Nigeria, Senegal, Togo)	1	12	1	2	2007	LA, PCR. CSF, S	1-59	2.00	NP	2198433.9	0.09097385	NP	6
Mwenda JM et al., 2019	2013	2013	1	AFR	West-Central Africa (Benin, Cameroon, Gambia, Ghana, Niger, Nigeria, Senegal, Togo)	1	12	1	2	2007	LA, PCR. CSF, S	1-59	4.00	NP	2276620.2	0.17569905	NP	6
Mwenda JM et al., 2019	2014	2014	1	AFR	West-Central Africa (Benin, Cameroon, Gambia, Ghana, Niger, Nigeria, Senegal, Togo)	1	12	1	2	2007	LA, PCR. CSF, S	1-59	7.00	NP	2358596	0.29678673	NP	6
Mwenda JM et al., 2019	2015	2015	1	AFR	West-Central Africa (Benin, Cameroon, Gambia, Ghana, Niger, Nigeria, Senegal, Togo)	1	12	1	2	2007	LA, PCR. CSF, S	1-59	2.00	NP	2444355.3	0.08182117	NP	6
Mwenda JM et al., 2019	2016	2016	1	AFR	West-Central Africa (Benin, Cameroon, Gambia, Ghana, Niger, Nigeria, Senegal, Togo)	1	12	1	2	2007	LA, PCR. CSF, S	1-59	4.00	NP	2533764.4	0.15786787	NP	6
Soeters HM et al., 2019	2015-2017	2016	1	AFR	MenAfriNet (Burkina Faso, Chad, Mali, Niger, Togo)	1	36	2	2	2008	CSF, PCR, LA, BC	0-59	85.63	13.27	15692300.81	0.54565612	0.15492958	7

Tagbo BN et al., 2019	2010	2010	1	AFR	Nigeria	1	12	2	1	2012	CSF, BC, G, LA, PCR, S	0-59	0.00	0.00	5,440,000.00	0	0	8
Tagbo BN et al., 2019	2011	2011	1	AFR	Nigeria	1	12	2	1	2012	CSF, BC, G, LA, PCR, S	0-59	0.00	0.00	5,440,000.00	0	0	8
Tagbo BN et al., 2019	2012	2012	1	AFR	Nigeria	1	12	2	1 + 2	2012	CSF, BC, G, LA, PCR, S	0-59	1.00	0.03	5,440,000.00	0.01838235	0.034	8
Tagbo BN et al., 2019	2013	2013	1	AFR	Nigeria	1	12	2	1 + 2	2012	CSF, BC, G, LA, PCR, S	0-59	2.00	0.07	5,440,000.00	0.03676471	0.034	8
Tagbo BN et al., 2019	2014	2014	1	AFR	Nigeria	1	12	2	1 + 2	2012	CSF, BC, G, LA, PCR, S	0-59	0.00	0.00	5,440,000.00	0	0	8
Tagbo BN et al., 2019	2015	2015	1	AFR	Nigeria	1	12	2	2	2012	CSF, BC, G, LA, PCR, S	0-59	0.00	0.00	5,440,000.00	0	0	8
Tagbo BN et al., 2019	2016	2016	1	AFR	Nigeria	1	12	2	2	2012	CSF, BC, G, LA, PCR, S	0-59	8.00	0.27	5,440,000.00	0.14705882	0.034	8
Von Gottberg A et al., 2012	2003-2009	2006	0	AFR	South Africa	2	84	1	2	1999	CSF, B, LA, G, PCR	0-59	349.00	66.31	35,420,157.50	0.98531465	0.19	7
Gentile et al., 2017	1992	1992	0	AMR	Bueno Aires, Argentina	2	12	2	1	1997	CSF, G, BC, B, A	0-59	10.00	0.57	182,234.00	5.4874502	0.05714286	7
Gentile et al., 2017	1993	1993	0	AMR	Bueno Aires, Argentina	2	12	2	1	1997	CSF, G, BC, B, A	0-59	19.00	0.57	182,234.00	10.4261554	0.03007519	7
Gentile et al., 2017	1994	1994	0	AMR	Bueno Aires, Argentina	2	12	2	1	1997	CSF, G, BC, B, A	0-59	10.00	0.57	182,234.00	5.4874502	0.05714286	7
Gentile et al., 2017	1995	1995	0	AMR	Bueno Aires, Argentina	2	12	2	1	1997	CSF, G, BC, B, A	0-59	9.00	0.57	182,234.00	4.93870518	0.06349206	7
Gentile et al., 2017	1996	1996	0	AMR	Bueno Aires, Argentina	2	12	2	1	1997	CSF, G, BC, B, A	0-59	12.00	0.57	182,234.00	6.58494024	0.04761905	7
Gentile et al., 2017	1997	1997	0	AMR	Bueno Aires, Argentina	2	12	2	1	1997	CSF, G, BC, B, A	0-59	3.00	0.57	182,234.00	1.64623506	0.19047619	7
Gentile et al., 2017	1998	1998	0	AMR	Bueno Aires, Argentina	2	12	2	1 + 2	1997	CSF, G, BC, B, A	0-59	7.00	0.57	182,234.00	3.84121514	0.08163265	7
Gentile et al., 2017	1999	1999	0	AMR	Bueno Aires, Argentina	2	12	2	2	1997	CSF, G, BC, B, A	0-59	0.00	0.00	182,234.00	0	0	7

Gentile et al., 2017	2000	2000	0	AMR	Bueno Aires, Argentina	2	12	2	2	1997	CSF, G, BC, B, A	0-59	0.00	0.00	182,234.00	0	0	7
Gentile et al., 2017	2001	2001	0	AMR	Bueno Aires, Argentina	2	12	2	2	1997	CSF, G, BC, B, A	0-59	1.00	0.00	157,607.00	0.63448958	0	7
Gentile et al., 2017	2002	2002	0	AMR	Bueno Aires, Argentina	2	12	2	2	1997	CSF, G, BC, B, A	0-59	0.00	0.00	157,607.00	0	0	7
Gentile et al., 2017	2003	2003	0	AMR	Bueno Aires, Argentina	2	12	2	2	1997	CSF, G, BC, B, A	0-59	0.00	0.00	157,607.00	0	0	7
Gentile et al., 2017	2004	2004	0	AMR	Bueno Aires, Argentina	2	12	2	2	1997	CSF, G, BC, B, A	0-59	0.00	0.00	157,607.00	0	0	7
Gentile et al., 2017	2005	2005	0	AMR	Bueno Aires, Argentina	2	12	2	2	1997	CSF, G, BC, B, A	0-59	2.00	0.00	157,607.00	1.26897917	0	7
Gentile et al., 2017	2006	2006	0	AMR	Bueno Aires, Argentina	2	12	2	2	1997	CSF, G, BC, B, A	0-59	0.00	0.00	157,607.00	0	0	7
Gentile et al., 2017	2007	2007	0	AMR	Bueno Aires, Argentina	2	12	2	2	1997	CSF, G, BC, B, A	0-59	0.00	0.00	157,607.00	0	0	7
Gentile et al., 2017	2008	2008	0	AMR	Bueno Aires, Argentina	2	12	2	2	1997	CSF, G, BC, B, A	0-59	2.00	0.00	157,607.00	1.26897917	0	7
Gentile et al., 2017	2009	2009	0	AMR	Bueno Aires, Argentina	2	12	2	2	1997	CSF, G, BC, B, A	0-59	0.00	0.00	157,607.00	0	0	7
Gentile et al., 2017	2010	2010	1	AMR	Bueno Aires, Argentina	2	12	2	2	1997	CSF, G, BC, B, A	0-59	0.00	0.00	165,638.00	0	0	7
Gentile et al., 2017	2011	2011	1	AMR	Bueno Aires, Argentina	2	12	2	2	1997	CSF, G, BC, B, A	0-59	0.00	0.00	165,638.00	0	0	7
Gentile et al., 2017	2012	2012	1	AMR	Bueno Aires, Argentina	2	12	2	2	1997	CSF, G, BC, B, A	0-59	0.00	0.00	165,638.00	0	0	7
Gentile et al., 2017	2013	2013	1	AMR	Bueno Aires, Argentina	2	12	2	2	1997	CSF, G, BC, B, A	0-59	0.00	0.00	165,638.00	0	0	7
Gentile et al., 2017	2014	2014	1	AMR	Bueno Aires, Argentina	2	12	2	2	1997	CSF, G, BC, B, A	0-59	3.00	0.00	165,638.00	1.81117859	0	7
Gentile et al., 2017	2015	2015	1	AMR	Bueno Aires, Argentina	2	12	2	2	1997	CSF, G, BC, B, A	0-59	5.00	0.00	165,638.00	3.01863099	0	7
Gentile et al., 2017	2016	2016	1	AMR	Bueno Aires, Argentina	2	12	2	2	1997	CSF, G, BC, B, A	0-59	2.00	0.00	165,638.00	1.2074524	0	7
Schossler JG et al., 2013	1999-2010	2005	0	AMR	Rio Grande do Sul, Brazil	2	144	1	2	1999	CSF, B	0-59	170.56	#REF!	9,659,430.00	1.77	#REF!	4
Zanella RC et al., 2011	2000-2002	2001	0	AMR	Brazil	2	36	1	2	1999	B, CSF, G, LA, PCR	0-59	54.00	NP	3,292,683.00	1.64	NP	9
Zanella RC et al., 2011	2003-2005	2004	0	AMR	Brazil	2	36	1	2	1999	B, CSF, G, LA, PCR	0-59	16.00	NP	3,200,000.00	0.5	NP	9
Zanella RC et al., 2011	2006-2008	2007	0	AMR	Brazil	2	36	1	2	1999	B, CSF, G, LA, PCR	0-59	19.00	NP	4,523,810.00	0.42	NP	9
Braikat M et al., 2012	2004-2009	2007	0	EMR	Morocco	2	72	2	1 + 2	2007	CSF, LA, G	0-59	105.00	42.00	17,848,638.00	0.58828018	0.4	10

Braikat M et al., 2012	2004	2004	0	EMR	Morocco	2	12	2	1	2007	CSF, LA, G	0-59	19.00	NP	2,974,773.00	0.6387042	NP	10
Braikat M et al., 2012	2005	2005	0	EMR	Morocco	2	12	2	1	2007	CSF, LA, G	0-59	29.00	NP	2,974,773.00	0.9748643	NP	10
Braikat M et al., 2012	2006	2006	0	EMR	Morocco	2	12	2	1	2007	CSF, LA, G	0-59	24.00	NP	2,974,773.00	0.80678425	NP	10
Braikat M et al., 2012	2007	2007	0	EMR	Morocco	2	12	2	1 + 2	2007	CSF, LA, G	0-59	21.00	NP	2,974,773.00	0.70593622	NP	10
Braikat M et al., 2012	2008	2008	0	EMR	Morocco	2	12	2	2	2007	CSF, LA, G	0-59	7.00	NP	2,974,773.00	0.23531207	NP	10
Braikat M et al., 2012	2009	2009	0	EMR	Morocco	2	12	2	2	2007	CSF, LA, G	0-59	5.00	NP	2,974,773.00	0.16808005	NP	10
Khalifa AB et al., 2011	1999-2006	2003	0	EMR	Monastir region, Tunisia	2	96	2	1 + 2	2011	CSF, LA	3-59	29.00	NP	417,639.16	6.94379324	NP	4
Khowaja AR et al., 2013	2008-2011	2010	1	EMR	Pakistan	1	42	2	2	2009	CSF, LA, PCR	0-59	26.00	7.00	1,889,596.80	1.37595491	0.26923077	8
Teleb N et al., 2013	2005	2005	0	EMR	Pakistan (Lahore, Karachi City)	1	12	1	1	2009	CSF, G, BC, PCR, LA	1-59	2.00	NP	2,551,775.74	0.07837679	NP	7
Teleb N et al., 2013	2006	2006	0	EMR	Pakistan (Lahore, Karachi City)	1	12	1	1	2009	CSF, G, BC, PCR, LA	1-59	10.00	NP	2,603,062.53	0.38416288	NP	7
Teleb N et al., 2013	2007	2007	0	EMR	Pakistan (Lahore, Karachi City)	1	12	1	1	2009	CSF, G, BC, PCR, LA	1-59	7.00	NP	2,655,787.68	0.26357529	NP	7
Teleb N et al., 2013	2008	2008	0	EMR	Pakistan (Lahore, Karachi City)	1	12	1	1	2009	CSF, G, BC, PCR, LA	1-59	0.00	NP	2,709,742.60	0	NP	7

Teleb N et al., 2013	2009	2009	0	EMR	Pakistan (Lahore, Karachi City)	1	12	1	2	2009	CSF, G, BC, PCR, LA	1-59	3.00	NP	2,677,680.63	0.11203726	NP	7
Teleb N et al., 2013	2010	2010	1	EMR	Pakistan (Lahore, Karachi City)	1	12	1	2	2009	CSF, G, BC, PCR, LA	1-59	2.00	NP	2,727,923.77	0.07331583	NP	7
Zaidi AK et al., 2010	2004-2005	2005	0	EMR	Pakistan (Karachi)	1	12	2	1	2009	CSF, LA, BC	0-59	28.00	0.00	2,333,333.33	1.2	0	7
Zaidi AK et al., 2010	2004-2005	2005	0	EMR	Pakistan (Hyderabad)	1	12	2	1	2009	CSF, LA, BC	0-59	17.00	0.00	222,855.00	7.6	0	7
Ceyhan M et al., 2014	2005-2010	2008	0	EUR	Turkey	1	72	2	1 + 2	2006	G, BC, CSF, PCR, A	1-59	50.95	1.30	13,267,349.00	0.3840104	NP	6
Ceyhan M et al., 2014	2011-2012	2011	1	EUR	Turkey	1	24	2	2	2006	G, BC, CSF, PCR, A	1-59	1.00	1.30	4,422,449.67	0.0226119	NP	6
Collin S et al., 2013	2009-2012	2011	1	EUR	UK (England & Wales)	1	48	2	2	1992	LA, PCR, CSF	0-59	15.00	1.00	38,333,333.00	0.03913	0.066667	4
Griffiths et al., 2011	2002-2008	2005	0	EUR	Belarus	2	84	1	2	2019	CSF, BC	1-59	30	NP	3,163,720.00	6.63775555	NP	7
Griffiths et al., 2011	2002	2002	0	EUR	Belarus	2	12	1	2	2019	CSF, BC	1-59	2.00	NP	451,960.00	0.44251704	NP	7
Griffiths et al., 2011	2003	2003	0	EUR	Belarus	2	12	1	2	2019	CSF, BC	1-59	6.00	NP	451,960.00	1.32755111	NP	7
Griffiths et al., 2011	2004	2004	0	EUR	Belarus	2	12	1	2	2019	CSF, BC	1-59	5.00	NP	451,960.00	1.10629259	NP	7
Griffiths et al., 2011	2005	2005	0	EUR	Belarus	2	12	1	2	2019	CSF, BC	1-59	7.00	NP	451,960.00	1.54880963	NP	7
Griffiths et al., 2011	2006	2006	0	EUR	Belarus	2	12	1	2	2019	CSF, BC	1-59	1.00	NP	451,960.00	0.22125852	NP	7
Griffiths et al., 2011	2007	2007	0	EUR	Belarus	2	12	1	2	2019	CSF, BC	1-59	5.00	NP	451,960.00	1.10629259	NP	7
Griffiths et al., 2011	2008	2008	0	EUR	Belarus	2	12	1	2	2019	CSF, BC	1-59	4.00	NP	451,960.00	0.88503407	NP	7
Griffiths et al., 2011	2002-2004	2003	0	EUR	Uzbekistan	2	36	1	2	2019	PCR, CSF	1-59	14.00	NP	9,486,453.00	0.14757887	NP	7
Hudeckova et al., 2010	1997-2007	2002	0	EUR	Slovakia	2	132	1	2	2000	CSF, LA	0-48	109.00	NP	2,849,000.00	3.82590383	NP	5
Levy C et al., 2014	2001-2012	2007	0	EUR	France	1	144	2	2	1992	CSF, PCR, BC	0-59	54.00	1.19	46,014,984.00	0.11735308	0.022	8
Batuwanthudawe et al., 2010	2004	2004	0	SEAR	Colombo, Srilanka	1	12	1	1	2008	BC, CSF, LA	0-59	54.00	3.00	179,103.00	30.1502	0.05555556	9
Jayaraman et al., 2018	2012-2013	2013	1	SEAR	India	1	12	2	2	2013	LA, PCR, CSF, B, BC	1-59	37.00	NP	638,491.67	5.79490727	NP	5
Cho HK et al., 2010	1996-2005	2001	0	WPR	Korea	2	120	2	2	2012	LA, CSF, BC, B	0-59	63.00	0.00	10925119	0.58	0.00	6
Greenhill et al., 2015	1996-2005	2001	0	WPR	Papua New Guinea	1	120	2	1	2008	G, BC, CC, CSF, S	0-59	148.00	NP	655,326.03	22.5841784	NP	7

Leung B et al., 2012	1995-2009	2002	0	WPR	Aukland, New Zealand	2	180	1	2	1994	CSF, BC, PCR	0-59	23.28	NP	1,411,155.00	1.65	NP	8
Nakamura R et al., 2013	2008	2008	0	WPR	Japan, Hokkaido	1	12	2	1	2011	BC, CSF, PCR	1-59	12.00	NP	206,910.00	5.8	NP	5
Nakamura R et al., 2013	2008	2008	0	WPR	Japan, Okinawa	1	12	2	1	2011	BC, CSF, PCR	1-59	3.97	NP	81,246.74	4.89	NP	5
Nakamura R et al., 2013	2009	2009	0	WPR	Japan, Hokkaido	1	12	2	1	2011	BC, CSF, PCR	1-59	13.00	NP	204,247.00	6.36	NP	5
Nakamura R et al., 2013	2009	2009	0	WPR	Japan, Okinawa	1	12	2	1	2011	BC, CSF, PCR	1-59	4.00	NP	81,798.00	4.89	NP	5
Nakamura R et al., 2013	2010	2010	1	WPR	Japan, Hokkaido	1	12	2	1	2011	BC, CSF, PCR	1-59	12.00	NP	203,366.00	5.9	NP	5
Nakamura R et al., 2013	2010	2010	1	WPR	Japan, Okinawa	1	12	2	1	2011	BC, CSF, PCR	1-59	7.00	NP	82,353.00	8.5	NP	5
Nakamura R et al., 2013	2007-2011	2009	0	WPR	Japan, Hokkaido	1	60	2	1	2011	BC, CSF, PCR	1-59	11.31	NP	203,366.00	5.56	NP	5
Nyambat B et al., 2011	2004-2005	2005	0	WPR	Vietnam, Ho Chi Minh City	2	24	2	1	2010	CSF, BC	0-59	15.00	1.00	66,666.67	22.5	0.06666667	9
Nyambat B et al., 2011	2004-2005	2005	0	WPR	Vietnam, Hanoi	2	24	2	1	2010	CSF, BC	0-59	10.00	0.00	102,040.82	9.8	0	9
Scott S et al., 2013	2002-2004	2003	0	WPR	Mongolia, Ulaanbaatar	1	36	2	1	2008	CSF, PCR, LA, BC	2-59	50.00	8.00	180,141.00	27.7560355	0.16	10
Scott S et al., 2013	2005-2007	2006	0	WPR	Mongolia, Ulaanbaatar	1	36	2	2	2008	CSF, PCR, LA, BC	2-59	8.00	1.00	180,141.00	4.44096569	0.125	10
Scott S et al., 2013	2008-2010	2009	0	WPR	Mongolia, Ulaanbaatar	1	36	2	2	2008	CSF, PCR, LA, BC	2-59	4.00	0.00	200,000.00	2	0	10
Wu et al., 2019	2006-2013	2010	1	WPR	China	1	96	2	2	N/A	B, CSF, PCR	0-59	10.00	NP	2,173,913.04	0.46	NP	5
Shinjoh et al., 2012	2009-2010	2010	0	WPR	Japan	2	24	2	2	2011	BC, PCR, CSF	1-59	85.00	15.30	1,164,383.56	7.3	0.18	6
Shinjoh et al., 2014	2011-2012	2012	1	WPR	Japan	2	24	2	2	2011	CSF, PCR, BC	0-48	82.00	NP	1,164,383.56	7.04235294	NP	9

Table S5: Quality Assessment Criteria

**Quality Criteria**		**Assessment**	**Score**	**Maximum Score**
Study Design	*Were the meningitis cases clearly defined: Clinical diagnosis and Laboratory confirmed diagnosis?*	Yes	1	**3**
		No	0	

	Were lumbar puncture/CSF consistently implemented across ALL study participants?	Yes	1	
		Ambiguous	0	
	Were inclusion and/or exclusion criteria for being in the study prespecified and applied uniformly to all participants?	Yes	1	
		No	0	
Were the limitations stated in the study?		Yes	2	**2**
		Ambiguous	1	
		No	0	
Sampling and Denominator	Was the sampling technique defined ?	Yes	2	**5**
		Unclear	1	
		No	0	
	Was the sample population given ?	Yes	1	
		Extrapolated	0	
	Was the sample appropriate for larger population ?	Yes	2	
		Unsure	1	
		No	0	
**Total (High: 7-10, Moderate: 4-6 , Low Quality: 0-3)**			**10**	

Table S6: Median, Minimum, and Maximum values of Incidence and CFR by WHO region

	Number of Studies	Median Incident Rate (Per 100,000 child-years)	Interquartile Range (IQR)	Minimum	Maximum	Median Case-Fatality Rate	Interquartile Range	Minimum	Maximum
Africa (AFR)	9	0.55	0.19-3.97	0.02	28.29	0.14	0.04-0.37	0.03	0.43
AMR (AMR)	3	1.77	1.27-4.94	0.42	10.43	0.06	0.05-0.11	0.03	0.36
Eastern Mediterranean (EMR)	5	0.61	0.22-1.03	0.07	7.60	0.33	0.30-0.37	0.27	0.40
Europe (EUR)	5	0.66	0.17-1.27	0.02	6.64	0.04	0.03-0.06	0.02	0.07
South East Asia (SEAR)	2	11.58	11.88-24.06	5.79	30.15	*	*	*	*
Western Pacific (WPR)	9	5.80	4.55-8.20	0.46	27.76	0.14	0.11-0.17	0.07	0.18
**World**	33	1.20	0.30-5.56	0.02	30.15	0.08	0.06-0.19	0.02	0.43

*Not enough data for accurate calculation

Figure S1: Funnel Plot Showing Distribution of Studies

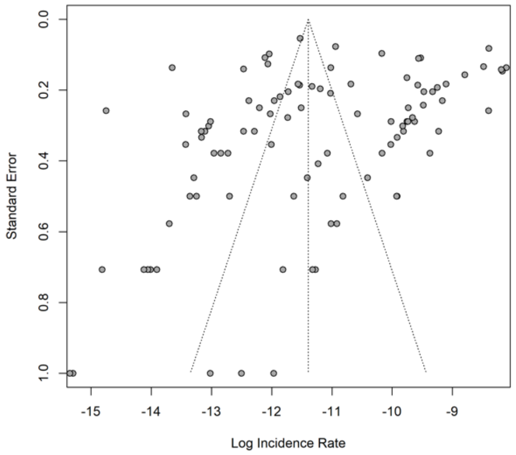

Figure S2: Meta-analysis Results

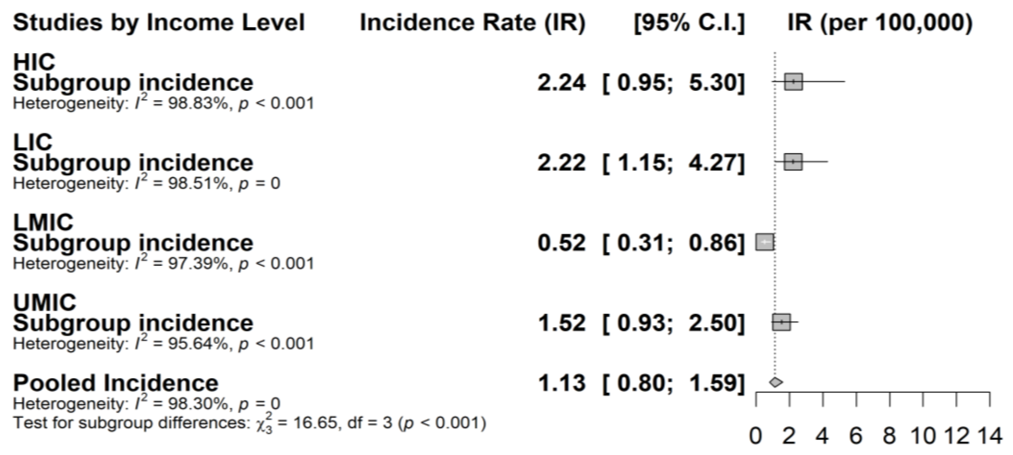

Incidence of Hib meningitis by income level of countries

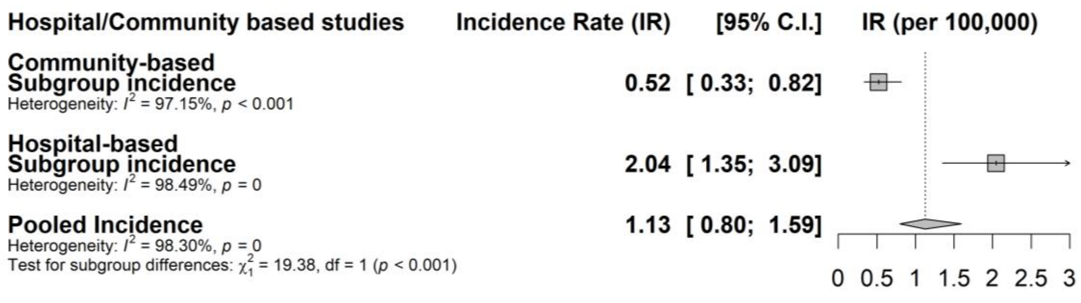

Incidence of Hib meningitis in Community-based studies and Hospital-based studies

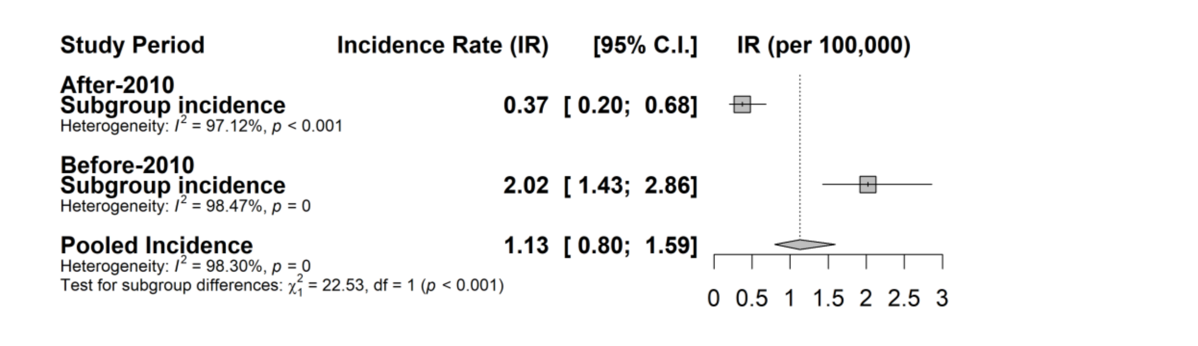

Incidence of Hib meningitis before 2010 and after 2010

Table S7: Vaccination Status of WHO Regions[18]

	Total Countries	Total Vaccination	% Vaccinated	Non Vaccinated	% Non vaccinated	Pre 2010	Since 2010	%Pre 2010	% Post 2010
Africa (AFR)	47	47	100.00%	0	0.00%	40	7	85.11%	14.89%
AMR (AMR)	54	35	64.81%	19	35.19%	33	1	94.29%	2.86%
Eastern Mediterranean (EMR)	23	21	91.30%	2	8.70%	16	5	76.19%	23.81%
Europe (EUR)	53	53	100.00%	0	0.00%	45	7	84.91%	13.21%
South East Asia (SEAR)	11	10	90.91%	1	9.09%	3	7	30.00%	70.00%
Western Pacific (WPR)	37	26	70.27%	11	29.73%	19	8	73.08%	30.77%
Total	225	192	85.33%	33	14.67%	156	35	81.25%	18.23%

Table S8: List of data sets with extrapolated population denominator

Author/Year of Publication	Study Period	Population Denominator
Ceyhan M et al., 2014	2011-2012	4,422,449.67
Greenhill et al., 2015	1996-2005	655,326.03
Jayaraman et al., 2018	2012-2013	638,491.67
Kaboré NF et al., 2012	2004-2005	166,109.81

Kaboré NF et al., 2012	2006-2008	280,515.46
Khalifa AB et al., 2011	1999-2006	417,639.16
Khowaja AR et al., 2013	2008-2011	1,889,596.80
Mwenda JM et al., 2019	2011	4644803.678
Mwenda JM et al., 2019	2012	4703752.899
Mwenda JM et al., 2019	2013	4943658
Mwenda JM et al., 2019	2014	5106123.716
Mwenda JM et al., 2019	2015	5246857.624
Mwenda JM et al., 2019	2016	5400276.569
Mwenda JM et al., 2019	2011	2123693.9
Mwenda JM et al., 2019	2012	2198433.9
Mwenda JM et al., 2019	2013	2276620.2
Mwenda JM et al., 2019	2014	2358596
Mwenda JM et al., 2019	2015	2444355.3
Mwenda JM et al., 2019	2016	2533764.4
Nakamura R et al., 2013	2008	81,246.74
Schossler JG et al., 2013	1999-2010	9,659,430.00
Soeters HM et al., 2019	2015-2017	15692300.81

Teleb N et al., 2013	2005	2,551,775.74
Teleb N et al., 2013	2006	2,603,062.53
Teleb N et al., 2013	2007	2,655,787.68
Teleb N et al., 2013	2008	2,709,742.60
Teleb N et al., 2013	2009	2,677,680.63
Teleb N et al., 2013	2010	2,727,923.77
Wu et al., 2019	2006-2013	2,173,913.04
Zaidi AK et al., 2010	2004-2005	2,333,333.33
